# Unraveling virus relationships by structure-based phylogenetic classification

**DOI:** 10.1093/ve/veaa003

**Published:** 2020-02-12

**Authors:** Weng M Ng, Alice J Stelfox, Thomas A Bowden

**Affiliations:** Division of Structural Biology, Wellcome Centre for Human Genetics, University of Oxford, Oxford OX3 7BN, UK

**Keywords:** virus, protein, structure, function, evolution

## Abstract

Delineation of the intricacies of protein function from macromolecular structure constitutes a continual obstacle in the study of cell and pathogen biology. Structure-based phylogenetic analysis has emerged as a powerful tool for addressing this challenge, allowing the detection and quantification of conserved architectural properties between proteins, including those with low or no detectable sequence homology. With a focus on viral protein structure, we highlight how a number of investigations have utilized this powerful method to infer common functionality and ancestry.

## 1. Introduction

Global genome sequencing initiatives have delivered abundant insights into the expansive viral biosphere (Li et al. 2015; Simmonds et al. 2017; Shi et al. 2018). Due to the complex relationship between gene sequence and protein function, using the information delivered by these analyses to understand virus pathobiology constitutes a recurrent challenge. Integrated structural analyses have proven an effective pathway by which to bridge this gap in knowledge, where the application of techniques in macromolecular structure determination, namely X-ray crystallography, nuclear magnetic resonance spectroscopy, and electron cryo-microscopy, have yielded molecular-level insights into virus structure and function (there were 10,958 entries in the Protein Data Bank using ‘viruses’ in the taxonomic search option on 24 January 2020).

Comparison of viral protein structures constitutes an important consideration when establishing the relatedness of proteins from genetically distinct viruses. When combined with modern sequence-based approaches (Simmonds et al. 2017; Wolf et al. 2018), structure-based phylogenetic analysis (SBPA) has proven to be an invaluable tool for drawing deep evolutionary links between proteins. Here, starting from a basic outline of known SBPA approaches, we discuss how the method has been applied to reveal unique insights into viral protein function and relatedness.

## 2. Understanding SBPA

SBPA relies on the availability of medium-to-high resolution (typically higher than 3 Å) structures, which can be compared in a pairwise manner to generate structural distances. Over the past decades, numerous tools have been developed to determine similarities between three-dimensional protein structures, including but not limited to HOMOLOGY (Rao and Rossmann 1973), Structural Homology Program (SHP) (Stuart et al. 1979), COMPARER (Sali and Blundell 1990), DALI (Holm and Sander 1993), CE (Shindyalov and Bourne 1998), MAMMOTH (Ortiz, Strauss, and Olmea 2002), FATCAT (Ye and Godzik 2004), SSM (Krissinel and Henrick 2004), Expresso/3D Coffee (Poirot et al. 2004; Armougom et al. 2006), Multiple Structural Alignment Algorithm (MUSTANG) (Konagurthu et al. 2006), SALIGN (Braberg et al. 2012), CLICK (Nguyen, Tan, and Madhusudhan 2011), and Homologous Structure Finder (HSF) (Ravantti, Bamford, and Stuart 2013). In this section, we briefly discuss the underlying process of structure-based alignments and phylogenetic analyses performed by programs such as SHP (Stuart et al. 1979), MUSTANG (Konagurthu et al. 2006), and HSF (Ravantti, Bamford, and Stuart 2013).

A pairwise structural alignment involves establishing the degree of equivalence between two superimposed structures. Conventionally, a measure of the root-mean-square distance between the corresponding Cα atoms of a pair of superimposed protein structures has been used to define structural similarity (Rossmann and Argos 1976). While such Cα alignment calculations work well for architecturally conserved proteins, they can be less adequate when comparing protein structures that are only remotely related. Therefore, pairwise structural alignments can be improved by added analysis of an array of parameters, such as solvent accessibility, dihedral angles, and local secondary structure. For example, MUSTANG (Konagurthu et al. 2006) aligns residues on the basis of similarity in residue–residue contact patterns and local structural topology, SHP (Stuart et al. 1979) utilizes the shape of the local polypeptide chain and a rotation function (Rossmann and Argos 1976) that infers the relative orientation of structures, and HSF (Ravantti, Bamford, and Stuart 2013) analyzes physiochemical and geometrical properties of the residues as well as local secondary structure.

Following pairwise structural alignments, HSF and MUSTANG (but not SHP) perform hierarchical categorization in a step termed ‘classification’. In HSF (Ravantti, Bamford, and Stuart 2013), the initial set of equivalent residues identified by pairwise comparison forms a common structural core between the two structures. Comparison of similarities between structural cores established from all of the pairwise alignments generates new combined cores (i.e. new sets of equivalent residues), which will subsequently be used to repeat this recursive alignment process until the pool of input structures under comparison is diminished. As each class grows to encompass more closely related structures, a full hierarchical classification is generated. Similarly, residue–residue similarity scores are generated by comparison of equivalent contiguous structural cores within MUSTANG (Konagurthu et al. 2006), where pairwise alignments are compared in the context of multiple structures to align classes.

Finally, the relatedness of protein structures can be calculated quantitatively and plotted as an unrooted tree (of unknown origin), reflecting the distances (branch length) between protein structures. SHP, MUSTANG, and HSF calculate so-called ‘evolutionary distances’ from similarity scores or probabilities of equivalence, as detailed in Bamford, Grimes, and Stuart (2005; SHP), Konagurthu et al. (2006; MUSTANG), and Ravantti, Bamford, and Stuart (2013; HSF). Subsequently, structure-based phylogenetic trees can be plotted from distance matrices and visualized using packages such as PHYLIP (Felsenstein 1989) and Dendroscope (Huson et al. 2007).

**Figure 1.**
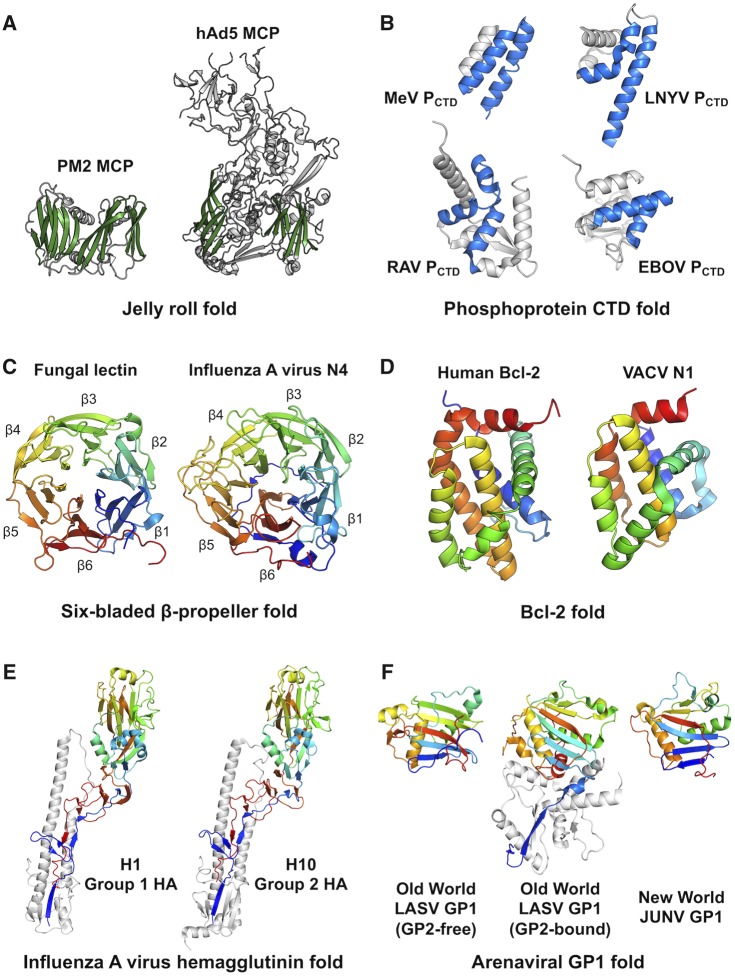
Representation of many of the protein folds discussed in this review. (A) The jelly roll fold. The structures of the major coat/capsid protein (MCP) of bacteriophage PM2 (PDB no. 2VVF) and human adenovirus 5 (1P30) are shown. The two four-stranded β-sheets that form the double jelly roll fold are colored green for clarity. (B) The P_CTD_. The structures of P_CTD_ from paramyxovirus (measles virus, MeV) (1OKS), rhabdoviruses (lettuce necrotic yellows virus, LNYV; and rabies virus, RAV) (3T4R and 1VYI), and filovirus (Ebola virus, EBOV) (3FKE) are shown. The α-helical core for each structure is colored blue, as determined by Martinez et al. (2013). (C) The six-bladed β-propeller fold. Structures of the fungal *Aleuria aurantia* lectin (1OFZ) and the influenza A virus N4 neuraminidase (2HTV) are shown and are colored as a rainbow from the N- (blue) to C-terminus (red). Each of the six ‘blades’ of the β-propeller is labeled accordingly (β1–β6). (D) The Bcl-2 fold. Structures of the human Bcl-2 protein (1G5M) and the vaccinia virus, VACV, N1 protein (2I39) are shown. The molecules are colored as in panel C. (E) The influenza A virus hemagglutinin (HA) fold. Structures of H1 (1RUZ) and H10 (4QY1) HAs, representing groups 1 and 2 HAs, respectively, are shown. The HA1 domains are colored as a rainbow from N- (blue) to C-terminus (red), while the HA2 domains are colored white. (F) The arenaviral GP1 fold. Structures of the OW LASV GP1, with and without GP2 (4ZJF and 5VK2), and the NW JUNV GP1 (5NUZ) are shown. GP1 molecules are colored as a rainbow ramped from blue (N-terminus) to red (C-terminus). GP2 is colored white for clarity. All structures are shown in cartoon representation.

While it is important not to discount the potential effects of structural determination (e.g. crystal packing) or inherent protein features (structural plasticity/flexibility) upon the calculation of these trees and the conclusions that can be drawn, in the following sections, we demonstrate how SBPA has been successfully used to relate commonalities in origin and function for several well-studied protein folds (Fig. 1).

## 3. Inferring evolutionary relationships of viruses and viral proteins

The identification of novel viruses continues to expand our appreciation of the virosphere, revealing a seemingly endless breadth of genomic diversity (Li et al. 2015; Shi et al. 2018). However, this breadth is not equally reflected in structure, where common protein folds are frequently identified amongst otherwise unrelated virus families (Luo et al. 2007; Abrescia et al. 2012; Cerny et al. 2014; Laanto et al. 2017; Ahola 2019). Such disproportion in magnitude of sequence versus structure variation may, in part, be attributed to stereochemical, geometric, and functional constraints on the folds of the protein. Indeed, while evolution of both gene and protein are restricted to maintain functionality, protein structure is additionally constrained to the approximately 2,000 unique folds predicted to exist amongst naturally occurring proteins (Bamford, Burnett, and Stuart 2002; Bamford 2003; Abrescia et al. 2012; Oksanen et al. 2012). While convergent evolution and gene transfer undoubtedly play an important role in the distribution of protein folds across the orders of life, the identification of conserved folds provides a unique opportunity to establish commonalities of function and even glean insights into evolutionary relationships amongst pathobiologically and genetically distinct viruses.

### 3.1 Using the jelly roll fold to decode evolutionary relationships

The single jelly roll fold was first observed over forty years ago in a structural study of the capsid of the single-stranded RNA tomato bushy stunt virus (Harrison et al. 1978), and was shown to comprise two four-stranded β-sheets that form the opposite sides of a β-barrel. The double jelly roll fold was later observed in the major coat/capsid protein (MCP) of the double-stranded DNA (dsDNA) human adenovirus (Roberts et al. 1986; Stewart et al. 1991), and is believed to have evolved via gene duplication and combination of single jelly roll proteins (Krupovic and Koonin 2017). The double jelly roll fold consists of a compact structure consisting of two β-barrels, each composed of eight anti-parallel β-strands arranged in two four-stranded sheets (Fig. 1A). Subsequent structural analysis of the MCP from the dsDNA bacteriophage, PRD1 (Benson et al. 1999), unexpectedly showed that the double jelly roll was also present in viruses with prokaryotic hosts. Since then, a myriad of dsDNA viruses and phages from diverse hosts and environments have been observed to present a coat protein with this same fold organization, including those that infect bacteria, archaea, green algae, and humans (i.e. bacteriophage PM2 (Abrescia et al. 2008), archaea *Sulfolobus* turreted icosahedral virus (Khayat et al. 2005), *Paramecium bursaria Chlorella* virus (Nandhagopal et al. 2002), and vaccinia virus (Bahar et al. 2011a), respectively).

The recent structural determination of the MCP from the ssDNA *Flavobacterium*-infecting, lipid-containing phage (FLiP) (Laanto et al. 2017), revealed that despite the difference in the type of genetic material packaged within the virion and the absence of significant sequence similarity to dsDNA viruses, the double jelly roll fold is also present in ssDNA viruses. Indeed, SBPA shows that FLiP MCP branches closely next to bacteriophage PM2 MCP (Laanto et al. 2017), supportive of an evolutionary relationship with the MCP members of the PRD1–adenovirus lineage (Abrescia et al. 2012; Ravantti, Bamford, and Stuart 2013). While a direct connection between sequences of FLiP MCP and previously characterized MCPs has proven elusive, genomic and metagenomic sequence analyses have linked FLiP to a group of ssDNA phages through their replication proteins (Yutin et al. 2018). These phages were shown to encode homologs of the FLiP MCP, suggestive of the existence of more ssDNA viruses with a double jelly roll-MCP (Yutin et al. 2018). These results demonstrate the power of complementary structure- and sequence-based approaches in drawing evolutionary links between diverse viruses.

The identification of shared structural and functional properties (e.g. common mode of capsid assembly and genome replication, respectively) across distinct MCP-bearing DNA viruses supports the existence of a common ancestor for the double jelly roll-MCP of PRD1–adenovirus members (Krupovic and Bamford 2008, 2011; Krupovic and Koonin 2017). Both SHP (Bamford, Grimes, and Stuart 2005; Abrescia et al. 2012) and HSF (Ravantti, Bamford, and Stuart 2013; Laanto et al. 2017) have been used to rationalize and quantify the proposed divergent evolution of this lineage, illustrating the power of SBPA in supporting and predicting evolutionary relationships. In line with the relationships inferred by SBPA (Abrescia et al. 2012; Ravantti, Bamford, and Stuart 2013; Laanto et al. 2017), a megataxonomic framework with a higher-level taxon (*Varidnaviria*) has recently been proposed to encompass a subset of eukaryotic and prokaryotic DNA viruses encoding vertical jelly roll-type MCPs (Koonin et al. 2019). Such a framework would potentially facilitate a rational structure-complemented approach for classifying newly identified and genetically diverse viruses bearing jelly roll MCPs.

### 3.2 Functional elaboration of a common protein core

A phosphoprotein (P) composed of a three-domain assembly (a disordered N-terminal domain, a central oligomerization domain, and a conserved C-terminal domain) plays essential roles in viral RNA synthesis across a number of negative-stranded RNA viruses from the order *Mononegavirales*, including members of the *Filoviridae*, *Paramyxoviridae*, and *Rhabdoviridae* families (Assenberg et al. 2010; Ivanov et al. 2010; Martinez et al. 2013). Although sequence homology of this protein across these families is low and in some cases undetectable (Delmas et al. 2010; Karlin and Belshaw 2012), structural analyses have revealed that the C-terminal domain (P_CTD_) of the molecule contains a common α-helical core (Fig. 1B), supporting an evolutionarily conserved function in mediating binding of P to the nucleocapsid protein (Green and Luo 2009; Ribeiro Ede et al. 2009). Investigations have shown that in addition to maintaining this conserved role, the P_CTD_ has acquired additional functionality through structural elaboration of the α-helical core. Indeed, the addition of five α-helices to the plant rhabdovirus P_CTD_, and α-helical and β-sheet subdomains to the filovirus P_CTD_ have both been shown to regulate viral transcription (Das et al. 1997) and facilitate additional RNA binding and immune evasion functionality (Leung et al. 2009, 2010). The structural elaboration of the evolutionarily conserved P_CTD_ core is reflected upon SBPA (Martinez et al. 2013), where P_CTD_ structures from each viral family classify to a single branch on the tree. In this case, SBPA provides an efficient means to visualize how a basic protein fold scaffold has been adapted to achieve different functionalities.

### 3.3 The β-propeller fold: A plastic architecture allows diverse functionality across domains of life

The six-bladed β-propeller fold (Fig. 1C) facilitates a diverse range of functionalities in an array of microorganisms, viruses, and higher eukaryotes (Gaskell, Crennell, and Taylor 1995; Chavas et al. 2005). A prominent example is the globular head domain of influenza virus neuraminidase (NA) (Shtyrya, Mochalova, and Bovin 2009), a six-bladed β-propeller sialidase, which catalyzes hydrolysis of the glycosidic linkage between sialic acid and the glycoconjugate presented on the host cell membrane to free the nascent virions from the infected cells (Crennell et al. 2000). This fold is also present in fucose-specific lectins (Wimmerova et al. 2003), soluble quinoprotein glucose dehydrogenases (Oubrie et al. 1999), and phytases (Kumar et al. 2017).

**Figure 2.**
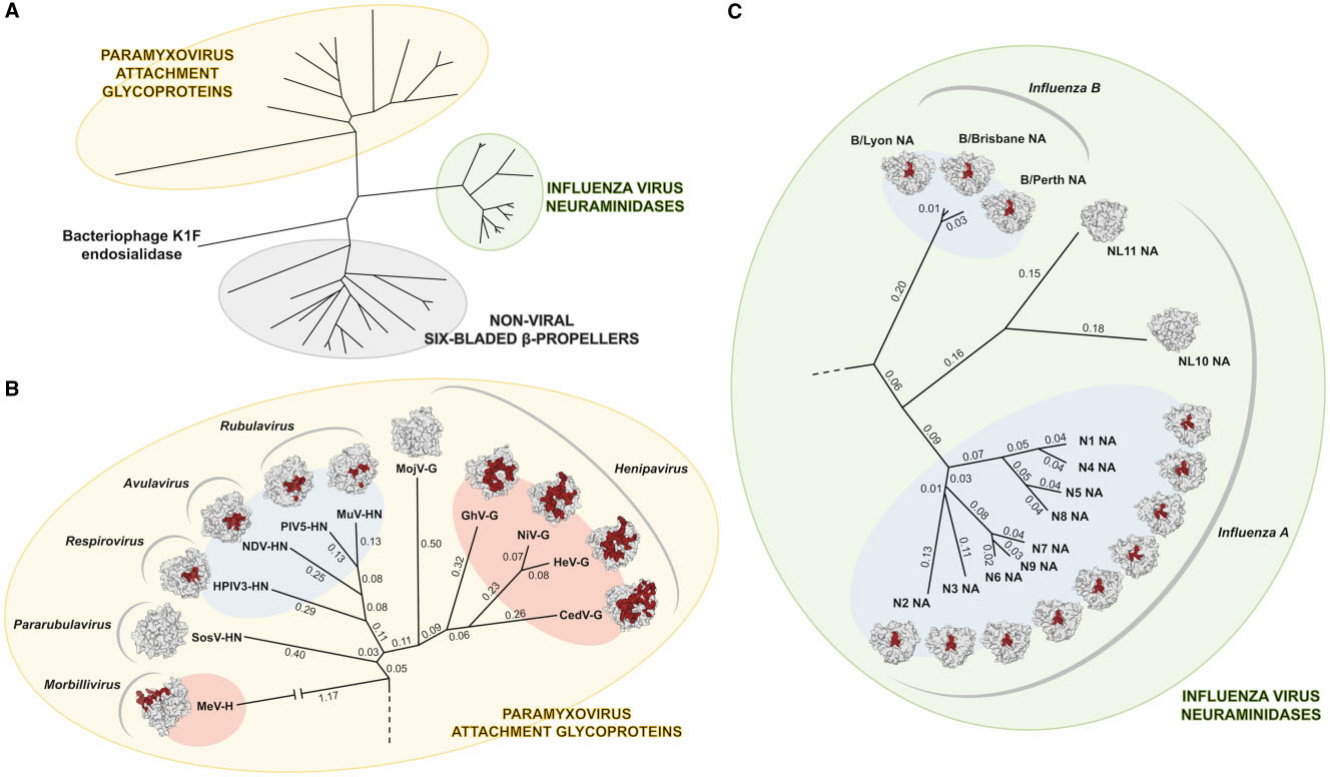
SBPA of six-bladed β-propeller structures. (A) Homologous six-bladed β-propeller structures were identified by application of the NDV-HN (PDB no. 1E8T) into the DALI server (Holm and Sander 1993). SBPA was subsequently performed with the following structures: MeV-H, measles virus hemagglutinin (2RKC); SosV-HN, Sosuga virus HN (6SG8); HPIV3-HN, human parainfluenza virus 3 HN (1V2I); NDV-HN; PIV5-HN, parainfluenza virus 5 HN (1Z4Y); MuV-HN, mumps virus HN (5B2C); MojV-G, Mòjiāng virus glycoprotein (5NOP); GhV-G, Ghana virus G (4UF7); NiV-G, Nipah virus G (2VSM); HeV-G, Hendra virus G (2X9M); CedV-G, Cedar virus G (6THB); influenza B virus NA molecules B/Perth (3K36), B/Lyon (4CPO), B/Brisbane (4CPL); influenza A virus NA molecules A/Vietnam N1 (2HTY), A/Tokyo N2 (1IVG), A/Missouri N3 (4HZV), A/Sweden N4 (2HTV), A/Alberta N5 (3SAL), A/England N6 (1V0Z), A/ALB N7 (4QN3), A/Ukraine N8 (2HT5), A/Australia N9 (7NN9), A/Guatemala NL10 (4FVK), A/Peru NL11 (4K3Y); *Salmonella* *typhimurium* sialidase (3SIL); *Trypanosoma rangeli* sialidase (1N1S); *Trypanosoma cruzi* trans-sialidase (1MR5); *Vibrio cholerae* sialidase (1KIT); *Streptococcus pneumoniae* NanA (2YA4); *Clostridium perfringens* NanI (2VK5); *S. pneumoniae* NanC (4YZL); *S. pneumoniae* NanB (2VW0); *Ruminococcus gnavus* intramolecular trans-sialidase (4X47); *Macrobdella decora* intramolecular trans-sialidase (1SLL); *Homo sapien* Neu2 (1SNT); *Micromonospora viridifaciens* sialidase (1EUT); *Aspergillus fumigatus* sialidase (2XCY); *Bacteroides thetaiotaomicron* sialidase (4BBW); *Pseudomonas aeruginosa* pseudaminidase (2W38); and bacteriophage K1F endosialidase (1V0F). An evolutionary distance matrix was calculated by SHP (Stuart et al. 1979) using pairwise structural superimposition of the six-bladed β-propeller structures and an unrooted tree was plotted with PHYLIP (Felsenstein 1989). Detailed views for SBPA of paramyxovirus attachment glycoproteins (yellow circle) and influenza virus neuraminidases (green circle) are presented in panels (B) and (C), respectively. (B and C) The β-propeller structures are shown in surface representation and colored white with the receptor-binding site colored red. β-propeller structures known to bind sialic acid are highlighted with a light blue background, while β-propeller structures that bind proteinaceous receptors are highlighted with a light red background. Note, the RBP of SosV is annotated as an HN glycoprotein, however, the receptor is currently unknown (Bowden et al. 2001; Chua et al. 2002; Johnson et al. 2019; Stelfox and Bowden 2019). Calculated evolutionary distances are indicated beside the branches.

The canonical six-bladed β-propeller fold is arranged as six β-sheets, each comprising four anti-parallel strands organized around a central axis (Fig. 1C). Previous studies have demonstrated that SBPA of proteins exhibiting this six-bladed β-propeller fold results in the bifurcation of viral and non-viral groups (Bowden et al. 2008). Interestingly, these two groups are linked by the endosialidase from the bacteriophage, K1F (Stummeyer et al. 2005) (Fig. 2A). Furthermore, the split of non-viral and viral six-bladed β-propellers coincides with the division of ASP-box-presenting and ASP-box-lacking β-propellers, respectively. The ASP-box, a short sequence and structural motif that occurs prominently in β-propellers (Quistgaard and Thirup 2009), is found in non-viral β-propellers (Roggentin et al. 1989; Crennell et al. 1993) and is absent from β-propellers from RNA viruses within the *Orthomyxoviridae* (Burmeister, Ruigrok, and Cusack 1992) and *Paramyxoviridae* families (Crennell et al. 2000). Given the presence of two ASP-box motifs in K1F endosialidase, it is possible that this six-bladed β-propeller evolved from other ASP-box presenting β-propellers that are non-viral in origin (Stummeyer et al. 2005).

### 3.4 A conserved protein architecture facilitates viral and antiviral functionality

Cellular proteins belonging to the B-cell lymphoma 2 (Bcl-2) family modulate apoptosis and hence, play an important role in clearing virus-infected cells (Youle and Strasser 2008). The Bcl-2 fold consists of six to seven amphipathic α-helices of varying lengths, which surround two central hydrophobic α-helices (Fig. 1D). To impede the host antiviral and innate immune response, some viruses, such as poxviruses, have evolved Bcl-2-like proteins (Douglas et al. 2007; Kvansakul et al. 2008; Maluquer de Motes et al. 2011), which exhibit anti-apoptotic functionality. Despite the overall dissimilarity in amino acid sequence and functionality, structural analyses have revealed that the cellular Bcl-2 and poxviral Bcl-2-like proteins exhibit remarkably similar folds (Bahar et al. 2011b).

Analogous to the split observed upon SBPA of viral and non-viral six-bladed β-propeller folds (Fig. 2A), SBPA of Bcl-2 and Bcl-2-like proteins (Graham et al. 2008; Bahar et al. 2011b; Neidel et al. 2015) reveals a rift between folds of cellular and poxviral origin. Close examination provides several insights into the relationships between these proteins. First, the SBPA reveals that despite low levels of sequence conservation (i.e. vaccinia virus VACV A49 protein shares only 8% sequence identity with myxoma virus MYXV M11 protein (Neidel et al. 2015)) and diverse functionality, poxviral Bcl-2-like proteins cluster closely together, suggestive that an ancestral poxvirus acquired a single Bcl-2 family gene and that duplication and diversification events during poxvirus evolution gave rise to structurally related proteins with different immunomodulatory functions (Graham et al. 2008; Bahar et al. 2011b; Neidel et al. 2015).

In addition, SBPA reveals that the structural relationship between poxviral Bcl-2-like proteins echoes their independently acquired functions. Indeed, VACV N1, which has both anti-apoptotic and anti-inflammatory functions (Maluquer de Motes et al. 2011), occupies a position on the phylogenetic tree intermediate between MYXV M11, which only exhibits anti-apoptotic functionality (Douglas et al. 2007), and VACV K7, B14, A46, and A52, which only exhibit anti-inflammatory activity (Bowie et al. 2000; Harte et al. 2003; Stack et al. 2005; Chen et al. 2008; Graham et al. 2008; Kalverda et al. 2009). This example demonstrates the power of SBPA to trace the evolution and diversification of protein functions from a common fold architecture.

## 4. Conceptualizing protein function through SBPA

As the repertoire of the PDB continues to expand, SBPA has been proven to be an increasingly useful method for rationalizing differences in protein functionality. Here, with a particular emphasis on viral receptor-binding proteins (RBPs), we demonstrate how SBPA provides insights into the means by which a common protein fold architecture can be adapted, modified, and elaborated to achieve differential functionality.

### 4.1 Paramyxovirus RBPs: Pathways to unique viral tropism characteristics

Paramyxoviruses exhibit some of the highest rates of cross-species transmission amongst RNA viruses (Kitchen, Shackelton, and Holmes 2011). This transmission potential is, in part, facilitated by the ability of the six-bladed β-propeller domain of the paramyxoviral RBP to productively interact with cell surface receptors that are conserved between different organisms (Eaton et al. 2006; Bowden et al. 2010; Thibault et al. 2017). Paramyxoviral RBPs categorize according to functionality into three groups: hemagglutinin–neuraminidase (HN), hemagglutinin (H), and attachment (G) glycoproteins. HN RBPs bind and hydrolyze sialic acid (Villar and Barroso 2006), H RBPs are presented by morbilliviruses and recognize SLAMF1 and nectin-4 receptors (Bieringer et al. 2013; Birch et al. 2013; Noyce, Delpeut, and Richardson 2013; Sakai et al. 2013; Melia et al. 2014; Ader-Ebert et al. 2015; Alves et al. 2015; Feng et al. 2016; Khosravi et al. 2016), and most henipaviral G RBPs are specific to ephrin receptors (Bonaparte et al. 2005; Negrete et al. 2005; Bowden, Jones, and Stuart 2011; Pernet, Wang, and Lee 2012; Rissanen et al. 2017).

SBPA of known paramyxoviral HN, H, and G RBP structures has shown that these viral proteins segregate according to receptor-specificity (Bowden et al. 2008; Lee et al. 2015; Rissanen et al. 2017; Stelfox and Bowden 2019; Pryce et al. 2020) (Fig. 2B). Indeed, despite sialic acid and ephrin-specific RBPs exhibiting low levels of sequence conservation within their respective classes (in some cases, less than 30%), these two groups of RBPs form distinct structural classes. Interestingly, although henipaviral G RBPs and morbilliviral H RBPs both bind proteinaceous receptors (ephrin and SLAMF1/nectin-4, respectively), they occupy unique branches. This observation is consistent with the contrasting modes of henipaviral and morbilliviral receptor recognition (Zeltina, Bowden, and Lee 2016) and supports the hypothesis that the departure of an ancestral virus from sialic acid to protein receptor specificity may have occurred more than once during paramyxovirus evolution (Bowden et al. 2008).

In addition, we note that the RBP from the henipavirus, Mòjiāng virus (MojV-G RBP) (Wu et al. 2014; Rissanen et al. 2017), falls outside the ephrin-specific G RBP grouping, and also away from H and HN RBPs (Fig. 2B). This observation is in-line with the absence of ephrin, SLAMF1, and sialic acid-binding motifs in MojV-G RBP (Zeltina, Bowden, and Lee 2016) and *in vitro* studies, which demonstrated that MojV undergoes a distinct host cell entry pathway (Rissanen et al. 2017). Similarly, the zoonotic paramyxovirus, Sosuga virus (SosV) (Albarino et al. 2014), also displays a distinct RBP structure, despite presenting some of the conserved residues known to be integral for sialic acid binding and hydrolysis. Indeed, unlike the HN RBPs of Newcastle disease virus (NDV) and mumps virus (MuV), for example, SosV-RBP presents a putative receptor-binding site incompatible with known modes of HN functionality (Stelfox and Bowden 2019). SBPA reflects the relatively close structural relationship, yet functional difference of SosV-RBP from HN RBPs (Fig. 2B), suggestive that SosV has only recently diverged from the well-established sialic acid binding and hydrolyzing functionality.

The identification of these structurally and functionally distinct RBPs both highlights the existence of novel host cell entry pathways that may be utilized by pathobiologically diverse paramyxoviruses and the power of SBPA in rationalizing and predicting host cell species tropism from paramyxoviral RBP structure alone.

### 4.2 Novel roles for structurally distinct influenza virus glycoproteins

Hemagglutinin (HA) (Fig. 1E) and neuraminidase (NA) (Fig. 1C) glycoproteins extend from the envelope surface of influenza virus and are responsible for negotiating host cell entry and egress, respectively. HA is responsible for both recognizing sialic acid during host cell attachment and facilitating fusion of the host and viral membranes, while NA is responsible for hydrolyzing sialic acid to free virus progeny from infected cells (Bouvier and Palese 2008; McAuley et al. 2019).

The paradigm shifting discovery of the HL17NL10 (HL, hemagglutinin-like; NL, neuraminidase-like) and HL18NL11 influenza A viruses from bats in Latin America (Tong et al. 2012, 2013), revealed the existence of influenza virus species lacking the canonical sialic acid binding and hydrolyzing functionalities of the HA and NA proteins (García-Sastre 2012; Li et al. 2012; Zhu et al. 2012; Sun et al. 2013; Tong et al. 2013; Moreira et al. 2016). Structural analysis of NL10 and NL11 revealed that in conjunction with a loss of some of the residues associated with sialidase activity, both presented a much wider putative sialic acid-binding site (Li et al. 2012; Tong et al. 2012, 2013; Zhu et al. 2012). Indeed, this differential functionality is reflected upon SBPA, which reveals that the structurally divergent NL10 and NL11 form a distinct branch that is nearly equidistant from the archetypal influenza A- and B-type NA structural groupings (Fig. 2C) (Li et al. 2012; Zhu et al. 2012).

**Figure 3.**
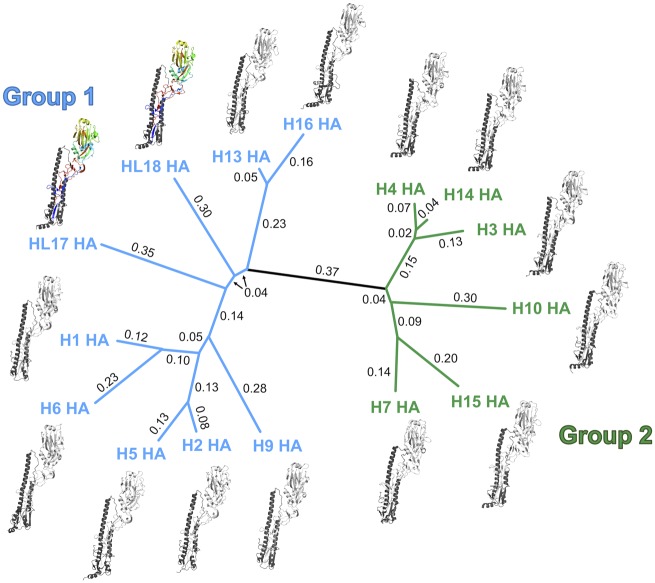
SBPA of the influenza A virus hemagglutinin (HA). Structures of influenza A virus HAs used were as follows: A/Brevig mission H1 (1RUZ), A/Japan H2 (2WRD), A/Finland H3 (2YP2), A/Czechoslovakia H4 (5XL5), A/Vietnam H5 (2FK0), A/New York H6 (4WSR), A/Italy H7 (1TI8), A/Hong Kong H9 (1JSD), A/Jiangxi-Donghu H10 (4QY1), A/Maryland H13 (4KPQ), A/Astrakhan H14 (3EYJ), A/Western Australia H15 (5TG8), A/Sweden H16 (4FIU), A/Guatemala HL17 (4I78), and A/Peru HL18 (4MC5). A pairwise evolutionary distance matrix was created using SHP (Stuart et al. 1979) and displayed as an unrooted phylogenetic tree using PHYLIP (Felsenstein 1989). The HA structures are shown in cartoon representation. HA1 domains for HL17 and HL18 are colored as a rainbow from the N- (blue) to C-terminus (red). HA1 domains for other HAs are colored white, while HA2 domains are colored dark gray. Branches indicating groups 1 and 2 influenza A virus HAs are colored blue and green, respectively. Calculated evolutionary distances derived from this analysis are indicated next to each branch.

While the receptor for HL18 has yet to be reported, the major histocompatibility complex class II human leukocyte antigen DR isotype (HLA-DR) has been identified as a receptor required for host cell attachment for HL17 (Karakus et al. 2019). Interestingly, both HL17 and HL18 do not exhibit great structural divergence from typical HA molecules, indicating that few alterations to the HA scaffold have been required to utilize this proteinaceous receptor (Karakus et al. 2019). Indeed, in contrast to the relatively large structural distances observed for NL10 and NL11 from classical influenza virus NAs (Fig. 2C), SBPA reveals that HL17 and HL18 locates closely to genetically related HA subtypes (Russell et al. 2008; Lazniewski et al. 2018) (Fig. 3). It is likely that the preservation of this close structural proximity, whilst adapting to different receptors, may reflect a functionally constrained requirement for HL17 and HL18 to also facilitate the conserved process of membrane fusion.

### 4.3 Discrete structural and functional states of the arenaviral GP1

Arenaviruses can be genetically and geographically categorized into Old World (OW) and New World (NW) serocomplexes (Emonet et al. 2009). Both OW and NW arenaviruses display a glycoprotein complex, GPC, which is composed of a retained stable signal peptide, GP1 attachment glycoprotein, and membrane-anchored GP2 fusion glycoprotein. Structural studies have shown that the arenaviral GP1 presents a compact α/β fold, and the GP2 forms a class I type fusion architecture (Bowden et al. 2009; Abraham et al. 2010; Igonet et al. 2011; Parsy et al. 2013; Cohen-Dvashi et al. 2015; Mahmutovic et al. 2015; Hastie et al. 2016, 2017; Li et al. 2016; Israeli et al. 2017; Shimon, Shani, and Diskin 2017; Zeltina et al. 2017; Clark et al. 2018; Pryce et al. 2018) (Fig. 1F). Similar to paramyxoviral RBPs, arenaviruses recognize a diverse array of receptors, including α-dystroglycan (OW and clade C NW) (Cao 1998; Spiropoulou et al. 2002), dendritic cell-specific intercellular adhesion molecule-3-grabbing non-integrin (OW) (Shimojima et al. 2012; Goncalves et al. 2013), lysosomal-associated membrane protein-1 (Lassa virus, LASV) (Jae et al. 2014), neuropilin-2 (Lujo virus, LUJV) (Raaben et al. 2017), tetraspanin CD63 (LUJV) (Raaben et al. 2017), and transferrin receptor 1 (clades B and D NW) (Radoshitzky et al. 2007; Abraham et al. 2009; Zong, Fofana, and Choe 2014). Non-covalently associated SSP–GP1–GP2 protomers are displayed on the mature virion surface as trimeric spikes (Li et al. 2016), where the location of the GP1 and GP2 have been determined by fitting a high resolution structure of a trimeric GP1–GP2 complex into a low resolution electron cryo-microscopy-derived map of the virion-displayed GPC (Hastie et al. 2017). Interestingly, it has been observed that the OW arenaviral GP1 presents different conformations in GP2-bound and unbound forms, where the GP2 is likely required to stabilize the conformation of GP1 observed on the mature GPC (Crispin et al. 2016; Hastie and Saphire 2018; Pryce et al. 2018).

**Figure 4.**
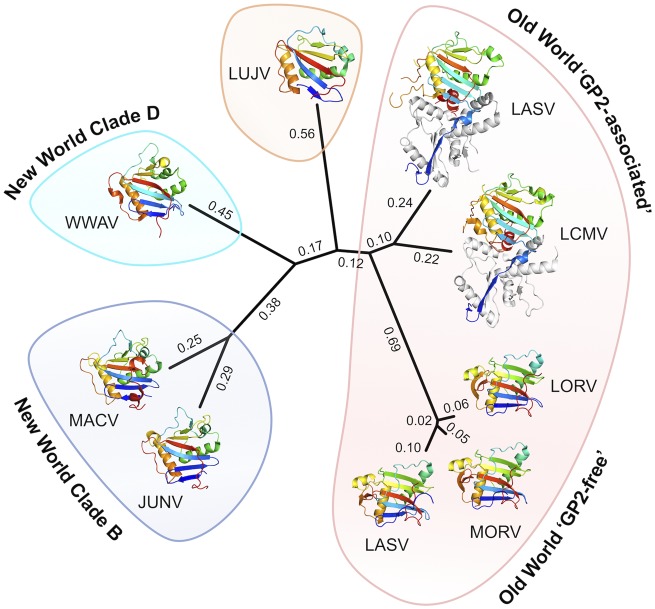
Structure-based phylogenetic tree showing the conformations adopted by arenaviral GP1 attachment glycoproteins. Structures of available arenavirus GP1 glycoproteins used were as follows: LORV, Loei River virus (PDB no. 6HJ6); LASV, Lassa virus (4ZJF and 5VK2); MORV, Morogoro virus (5NFF); LCMV, Lymphocytic choriomeningitis virus (5INE); LUJV, Lujo virus (6GH8); WWAV, Whitewater Arroyo virus (6HJ5); MACV, Machupo virus (2WFO); and JUNV, Junín virus (5NUZ). For 5VK2, 5INE, 6GH8, and 5NUZ, all chains not comprising GP1 molecules (e.g. GP2, receptor, and antibody fragments) were removed prior to structural alignment. A pairwise evolutionary distance matrix was created using SHP (Stuart et al. 1979) and displayed as an unrooted phylogenetic tree using PHYLIP (Felsenstein 1989). GP1 structures are shown in cartoon representation and colored as a rainbow from the N- (blue) to C-terminus (red). Although the GP2 component of the GPC was not included in the structure comparison, it is shown and colored as a white cartoon for clarity. Calculated evolutionary distances derived from this analysis are indicated next to each branch.

A recent structural study utilized SBPA to classify the conformational states of OW and NW arenaviral GP1 glycoproteins and relate the potential physiological roles of these discrete structural classes to differential antigenicity (Pryce et al. 2018). This analysis revealed that GP1 glycoproteins broadly divide into two branches (Fig. 4) dependent on their OW or NW lineage. Close examination of the structure-based phylogenetic tree reveals that OW arenaviral GP1 glycoproteins subdivide into two separate structural classes: one class comprises structures of OW GP1s solved in association with a cognate OW GP2 (GP2-bound), and the other contains GP1 structures solved in the absence of a GP2 (GP2-free). The existence of two discrete OW arenaviral GP1 conformations highlights the potential for antigenic deconfiguration of the molecule once shed from the GPC during host cell entry (Branco et al. 2010; Hastie et al. 2017; Pryce et al. 2018), and provides a structure-based rationale for how shed OW GP1 may serve as an immunological decoy that contributes to the often ineffective humoral immune response observed early in the infection (Fisher-Hoch et al. 2000; Lukashevich et al. 2008; Branco et al. 2010). The hypothesis that GP2-free OW GP1 glycoproteins present epitopes not represented on the mature GPC is supported by the absence of an effective humoral immune response upon immunization with recombinant OW GP1 (Borenstein-Katz et al. 2019).

Inspection of the SBPA reveals that the GP1 structures from NW Machupo virus (MACV), Junín virus (JUNV), and Whitewater Arroyo virus (WWAV) separate into two branches according to respective clades B and D classification (Pryce et al. 2018). Due to the current paucity of GP2-bound NW GP1 structures, it is unknown whether the NW GP1 exhibits the same structural plasticity as OW GP1s. However, this has been hypothesized to be unlikely as GP2-free NW GP1 elicits a neutralizing antibody immune response following immunization in animal models, is recognized by vaccine-elicited neutralizing antibodies, and recognizes the host transferrin receptor 1 (Abraham et al. 2010; Mahmutovic et al. 2015; Zeltina et al. 2017; Clark et al. 2018; Borenstein-Katz et al. 2019).

Strikingly, we note that the GP2-free structure of the GP1 glycoprotein from LUJV, a pathogenic OW arenavirus (Briese et al. 2009), presents a dramatically different architecture to previously reported NW and OW arenaviral GP1 structures (Cohen-Dvashi, Kilimnik, and Diskin 2018). LUJV GP1 was crystallized in complex with the neuropilin-2 receptor, indicative that the observed conformation is likely representative to that observed on the mature GPC. In line with the unique receptor tropism characteristics and structure of LUJV GP1, which includes features observed in both NW arenaviral GP1s and OW arenaviral GP2-bound GP1s (Cohen-Dvashi, Kilimnik, and Diskin 2018), SBPA reveals that the glycoprotein falls outside both NW and OW classes (Fig. 4). Indeed, the near-equal structural distance of LUJV GP1 from both NW and OW GP1 classes is supportive of the hypothesis that the glycoprotein structurally and functionally diverged early in the evolutionary bifurcation of OW and NW arenaviruses.

## 5. Conclusions

In light of the expansive genomic diversity revealed by viral surveillance initiatives (Li et al. 2015; Shi et al. 2018), SBPA has proven to complement sequence analysis tools as a *tour de force* method for gleaning evolutionary relationships between genetically distinct viruses. In our review, we have highlighted many instances of how this has been achieved. For example, we discuss how this method has been previously used to show the way the double jelly roll fold, which is found in many viral coat proteins, has evolved to facilitate the assembly of genetically distinct viruses (Ravantti, Bamford, and Stuart 2013; Laanto et al. 2017). Moreover, we have demonstrated how SBPA provides a blueprint for understanding the means by which bacteriophage endosialidase scaffolds may have evolved from six-bladed β-propeller proteins of non-viral origin (Fig. 2A). Importantly, however, we note that the presented case-studies are not exhaustive and that there are other biological systems amenable to investigation by this powerful method. For instance, a series of SBPA-based studies focused on the structurally conserved common core of right-handed RNA and DNA polymerases have successfully recapitulated the relationships of the six established right-handed polymerase families in a single phylogeny (Cerny et al. 2014; Monttinen et al. 2014; Cerny et al. 2015; Jacome et al. 2015).

It seems likely that SBPA will continue to play an important role in characterizing and rationalizing the pathobiological features of newly emerging viruses. Indeed, we have shown that this is possible for emerging paramyxoviruses, where the receptor-binding domain, an important determinant of host cell tropism, structurally classifies according to receptor specificity (Rissanen et al. 2017; Stelfox and Bowden 2019; Pryce et al. 2020) (Fig. 2B). Similarly, reflective of their sialic acid independent functionality, NL glycoproteins from bat borne influenza viruses form a structural class that is distinct from archetypal NA glycoproteins (Fig. 2C). Analogously, SBPA of the RNA virus phosphoprotein CTD core (Martinez et al. 2013) and Bcl-2/Bcl-2-like proteins (Graham et al. 2008; Bahar et al. 2011b; Neidel et al. 2015) provides a context for how a common protein fold may be elaborated to achieve distinct functionality.

**Figure 5.**
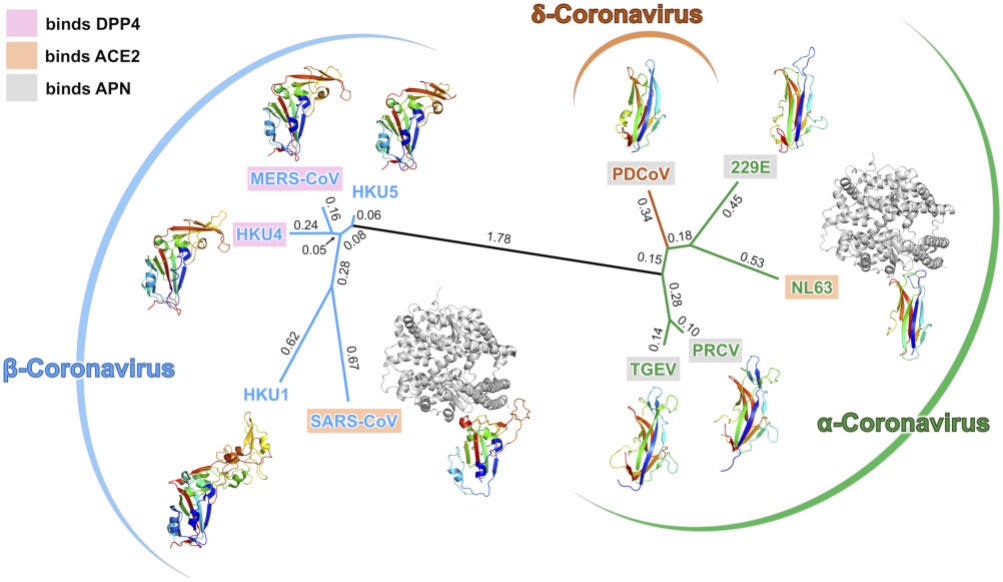
SBPA of coronavirus S1-CTD glycoproteins. Structures of coronavirus S1-CTD glycoproteins used were as follows: human coronavirus HKU1 (PDB no. 5GNB); HKU4 (4QZV); HKU5 (5XGR); Middle East respiratory syndrome coronavirus, MERS-CoV (4ZPW); severe acute respiratory syndrome coronavirus, SARS-CoV (3D0I); transmissible gastroenteritis coronavirus, TGEV (4F2M); porcine respiratory coronavirus, PRCV (4F5C); human coronavirus NL63 (3KBH); human coronavirus 229E (6ATK); porcine deltacoronavirus, PDCoV (6BFU). All chains not comprising S1-CTD (e.g. receptor and antibody fragments) were removed prior to structural alignment. A pairwise evolutionary distance matrix was created using SHP (Stuart et al. 1979) and displayed as an unrooted phylogenetic tree using PHYLIP (Felsenstein 1989). Branches corresponding to alpha-, beta-, and delta-coronaviruses are colored green, blue, and brown, respectively. S1-CTD structures are shown in cartoon representation and colored as a rainbow from the N- (blue) to C-terminus (red). Although ACE2 was not included in the structure comparison, it is shown and colored here as a white cartoon. This analysis demonstrates that although SARS-CoV and NL63 S1-CTDs utilize the same receptor (Li 2005, 2008; Wu et al. 2009; Song et al. 2018), the structures classify according to genetic relationship with other coronavirus S1-CTDs, rather than receptor tropism characteristics. The viruses are color-coded according to their receptor usage: pink for dipeptidyl peptidase 4 (DPP4); orange for angiotensin converting enzyme 2 (ACE2); and gray for aminopeptidase N (APN). Calculated evolutionary distances derived from this analysis are indicated next to each branch.

SBPA can also lead to structure-guided hypotheses of whether the glycoproteins displayed by emerging viruses may be capable of misleading the antibody-mediated immune response through the formation of structural classes that are distinct from those presented on the mature virion (Pryce et al. 2018) (Fig. 4). However, it should be noted that the intricacies of viral protein function may not always be unambiguously represented by means of SBPA. Indeed, the absence of receptor-specific clustering for C-terminal domains of the S1 receptor-binding subunit (S1-CTD) from the family *Coronaviridae* (Fig. 5) suggests a complex evolutionary history for these viruses, which may have involved the switch to the same host cell receptor (e.g. ACE2) on multiple occasions (Li 2005, 2008, 2015; Wu et al. 2009; Song et al. 2018). Thus, where SBPA is feasible, it constitutes an important resource in the toolkit of strategies that can be used for characterizing, relating, and predicting the functionality of proteins presented by biomedically important viruses. As such, we suggest that future work focused on comparison and development of SBPA methods will be of great value for the structural virology community and efforts to understand virus pathobiology at a holistic level.
